# Intermediate Muscle Length and Tendon Vibration Optimize Corticospinal Excitability During Knee Extensors Local Vibration

**DOI:** 10.3389/fphys.2018.01266

**Published:** 2018-09-05

**Authors:** Robin Souron, Marie Oriol, Guillaume Y. Millet, Thomas Lapole

**Affiliations:** ^1^Univ Lyon, UJM Saint-Etienne, Laboratoire Interuniversitaire de Biologie de la Motricité, EA 7424, F-42023, Saint-Étienne, France; ^2^Human Performance Laboratory, Faculty of Kinesiology, University of Calgary, Calgary, AB, Canada

**Keywords:** local vibration, transcranial magnetic stimulation, motor-evoked potentials, knee extensors, muscle length, vibration site

## Abstract

While local vibration (LV) has been recently proposed as a potential modality for neuromuscular conditioning, no practical recommendations to optimize its effects have been published. Because changes in corticospinal excitability may reflect at which degree the neuromuscular function is modulated during LV exposure, this study investigated the effects of muscle length and vibration site on LV-induced on motor evoked potentials (MEPs) changes. Twenty-one subjects participated in a single session in which MEPs were evoked on the relaxed knee extensors (KE) during three conditions, i.e., no vibration (CON), muscle (VIB_MU_), and tendon vibration (VIB_TD_). Three muscle lengths were tested for each condition, i.e., short/intermediate/long KE muscle length. Both VIB_MU_ and VIB_TD_ significantly increase MEPs compared to CON. Higher increases (*P* < 0.001) were found for VIB_TD_ compared to VIB_MU_ for vastus lateralis (mean increases of the three angles: +241% vs.+ 148%), vastus medialis (+273% vs. + 180%) and rectus femoris muscles (+191% vs. +141%). The increase in MEPs amplitude was higher (*p* < 0.001) at an intermediate (mean pooled increase for VIB_TD_ and VIB_MU_: +265%, +290%, and +212% for VL, VM, and RF, respectively) compared to short (+136%, + 144%, and + 127%) or long (+ 184%, + 246% and + 160%) muscle lengths. These results suggest that LV should be applied to the tendon at an intermediate muscle length to optimize the acute effects of LV on the KE neuromuscular function.

## Introduction

When local vibration (LV) is acutely applied for 20–30 min to a muscle or a tendon, fatigue, i.e., a decrease in maximal-force generating capacity, is commonly reported (Kouzaki et al., 2000; Konishi et al., 2002; Jackson and Turner, 2003; Yoshitake et al., 2004; Shinohara et al., 2005; Ushiyama et al., 2005; Richardson et al., 2006; Herda et al., 2009; Saito et al., 2016a; Souron et al., 2017b). It is suggested to be principally due to a reduction of maximal central drive (Jackson and Turner, 2003; Ushiyama et al., 2005; Souron et al., 2017b) related to the strong activation of muscle spindles Ia afferents during LV (Burke et al., 1976a). Since LV stimuli is a significant neuromuscular workload that induces fatigue, it is not surprising that when repeated, it could trigger long-term adaptations leading to improved neuromuscular function (Souron et al., 2017c), as already reported for traditional training that involved a variety of voluntary dynamic exercises, e.g., resistance training or cycling (Burtin et al., 2012; Gathercole et al., 2015). Indeed, increased maximal-force generating capacity was recently reported after LV training performed on relaxed muscles over 14 to 180 days (Lapole and Pérot, 2010, 2012; Iodice et al., 2011; Lapole et al., 2013; Tankisheva et al., 2015; Souron et al., 2017a,d). However, LV as a modality for neuromuscular conditioning remains relatively under-investigated and, more importantly, studies that have addressed this question have used a wide variety of protocols [see Souron et al. (2017c) for a review]. In particular, the influence of training (e.g., duration, number of sessions) and vibration (e.g., frequency, amplitude, site of application, relaxed vs. contracted muscle) characteristics are not well understood. As a result, it is currently difficult to suggest practical recommendations that would optimize the effects of LV and considerable work remains to help establishing research-based guidelines for LV, especially with the perspective of clinical use [see Souron et al. (2017c) for a review].

Modulations of corticospinal excitability during short-time LV (4–60 s) has been reported, e.g., an increase in motor evoked potentials (MEPs) size in extensor (Kossev et al., 1999; Siggelkow et al., 1999; Kossev et al., 2001) and flexor carpi radialis (Rosenkranz et al., 2003; Steyvers et al., 2003b) as well as soleus (Lapole et al., 2015a,b) and gastrocnemius medialis muscles (Lapole et al., 2015b). Such an increase in MEPs during LV exposure is thought to be directly mediated by the vibration-induced discharge of Ia afferents (Roll et al., 1989). If MEPs modulation during LV reflects how strong Ia afferents are activated, and assuming that acute and chronic effects of LV are directly related to this activation (Souron et al., 2017c), investigating the influence of vibration characteristics on MEPs amplitude may help to define optimal LV characteristics. While this has never been investigated so far in the context of vibration, it may also help to discriminate if Ia afferent feedback during LV is the same for all muscles/motoneurones for an identical muscle group, i.e., knee extensors (KE).

Local vibration frequency has been shown to be an important parameter in the modulation of corticospinal responses (Siggelkow et al., 1999; Steyvers et al., 2003b; Lapole et al., 2015b). Yet other parameters such as the vibration site or the initial state of the muscle may also influence Ia afferents activation and corticospinal excitability during LV. Indeed, tendon vibration is known to facilitate the occurrence of the tonic vibration reflex when compared to muscle vibration (De Gail et al., 1966; Eklund and Hagbarth, 1966), probably because of a greater discharge of Ia afferents and/or the activation of other endings than the primary ones, e.g., type II afferents (Burke et al., 1976b). Moreover, Ia afferents have been demonstrated to be more responsive to LV when the muscle is stretched and during voluntary isometric contractions (Burke et al., 1976a; Burke, 1980), probably due to the presence of alpha–gamma co-activation (Vallbo, 1974) and/or superior transmission through the musculo-tendinous structures (Burke et al., 1976a), respectively.

Therefore, in line with our recent works that investigated the acute (Souron et al., 2017b) and chronic effects (Souron et al., 2017a) of LV on neuromuscular function of the KE, the present study aimed to investigate the effects of muscle length and vibration site (i.e., muscle vs. tendon) on LV-induced changes in corticospinal excitability of the KE. We hypothesized that the increase in MEPs amplitude during LV would be higher at a knee angle corresponding to a lengthened quadriceps muscle because of the increased responses of Ia afferents at long muscle length (Burke et al., 1976b). We also hypothesized that the magnitude of the increase in MEPs would be greater when vibration is locally applied to the tendon compared to applied to the muscle because of a greater afferent discharge (Burke et al., 1976b).

## Materials and Methods

### Subjects

Twenty-one healthy subjects (16 men and 5 women, age: 24 ± 3 year; height: 177 ± 9 cm; weight: 74 ± 15 kg) participated in this study. They reported to be recreationally active, i.e., approximately 1–2 h of physical activity 3 times per week (Kean et al., 2006), and perform mainly team sport activities (e.g., football, basketball). This study was carried out in accordance with the recommendations of the local ethics committee (CPP Sud Est I) with written informed consent from all subjects. All subjects gave written informed consent in accordance with the Declaration of Helsinki. The protocol was approved by the local ethics committee (NCT02929004). All subjects had no contraindications to TMS (Rossi et al., 2011) and in particular no acute or chronic neurological disorders and trauma. Participants were advised to avoid strenuous exercise and to maintain their sleeping, eating and drinking habits for ≥ 24 h. However, they were specifically instructed to avoid caffeine, alcohol or energy drinks for ≥ 12 h before experiments.

### Experimental Protocol

**FIGURE 1:**
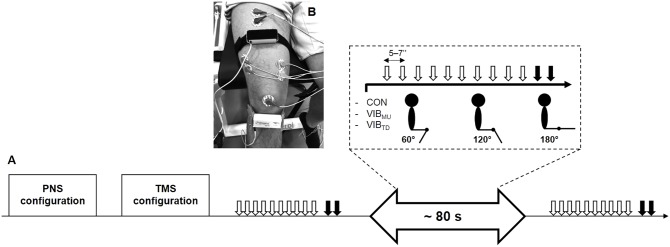
Overview of the experimental protocol **(A)**. Series of ten transcranial magnetic (white arrows) and two peripheral nerves stimulations (black arrows) were performed at three different muscle lengths (i.e., corresponding to knee angles of 60, 120, and 180°) for the three conditions, i.e., no vibration (CON), muscle vibration (VIB_MU_), and tendon vibration (VIB_TD_). Illustration of the position of the vibratory devices on the rectus femoris muscle and the infrapatellar tendon at a knee angle of 120° **(B)**.

**FIGURE 2:**
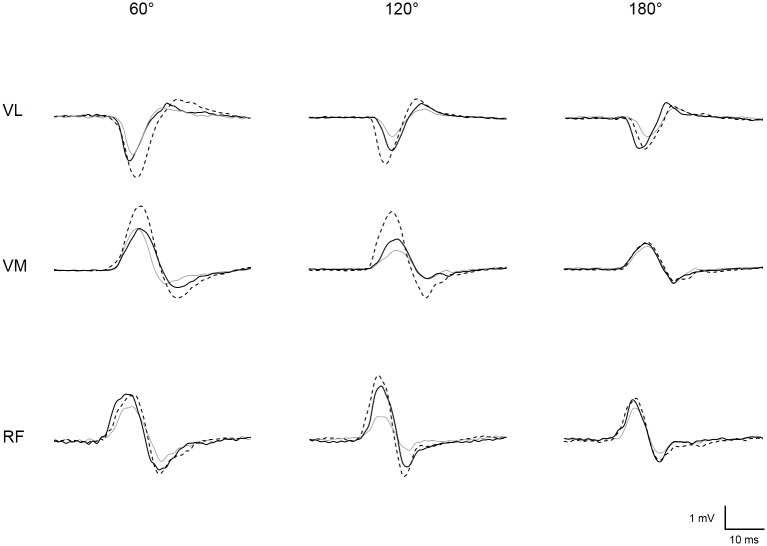
Representative trace of MEPs recorded for vastus lateralis (VL), vastus medialis (VM), and rectus femoris (RF) at each muscle length (i.e., knee angle of 60, 120, and 180°) when vibration was locally applied on the muscle (solid black line) or the tendon (dotted black line) or when vibration was turned off, i.e., control condition (solid gray line).

The experimental protocol is illustrated in **Figure 1A**. Subjects were seated on an isometric dynamometer (LegControl, Matsport, Saint Ismier, France) for the entire duration of the testing session. First, optimal positions and intensities of stimulation were determined for peripheral nerve (PNS) and transcranial magnetic stimulation (TMS) techniques (see below). Then, series of ten TMS and two PNS pulses were delivered on the relaxed muscle during three conditions, i.e., no vibration (CON), muscle vibration (VIB_MU_) and tendon vibration (VIB_TD_). Each condition was performed at three different muscle lengths corresponding to knee angles of 60, 120, and 180° (180° is full extension). The intermediate knee angle at 120° was first selected to ensure a minimal muscle passive tension and then spindle sensitivity since it corresponds to an angle that is slightly above the slack angle for quadriceps muscles (Xu et al., 2016). The 60°-knee angle was selected as a long muscle length where high degree of passive muscle tension is produced (Xu et al., 2016). Finally, a condition with the leg completely extended (i.e., 180°) was useful to refer to previous LV studies that applied LV sessions on the shortened knee extensor muscles (Iodice et al., 2011; Tankisheva et al., 2015). Hence, a total of nine combinations (3 conditions × 3 knee angles) were tested. Rest periods of 1 min were provided between testing series. The order of the conditions as well as the tested knee angles were randomized. The duration of the vibration was ∼80 s, i.e., the time necessary to evoke the series of ten MEPs and two maximal M-waves (M_max_, see below) with an interval of 5–7 s between each stimulation. To control for possible changes in basal corticospinal excitability throughout the experimental protocol, one series of ten MEPs and two M_max_ was performed without vibration before and just after the end of the protocol at a knee angle of 120°. The size of the MEPs and M_max_ were measured as the peak-to-peak amplitude of the non-rectified response and averaged for each muscle. A representative trace of MEPs for each condition and each knee angle is displayed in **Figure 2**. Mean MEP amplitudes for each series were normalized to mean M_max_ recorded on the same series and muscle.

### Electromyographic Activity

Electromyographic (EMG) signals were recorded from rectus femoris (RF), vastus lateralis (VL), and vastus medialis (VM) muscles with pairs of self-adhesive surface electrodes (Meditrace 100; Covidien, Mansfield, MA, United States) in bipolar configuration with a 30-mm interelectrode distance. According to SENIAM recommendations, RF electrodes were placed at 50% on the line from the anterior spina iliaca superior to the superior part of the patella; VL electrodes at 2/3 on the line from the anterior spina iliaca superior to the lateral side of the patella; VM electrodes at 90% on the line between the anterior spinac iliaca superior and the joint space in front of the anterior border of the medial ligament. Low impedance (<5 kΩ) between electrodes was obtained by shaving and gently abrading the skin and then cleaning it with isopropyl alcohol. Signals were amplified with an octal bio-amplifier (ML138, ADInstruments, Bella Vista, NSW, Australia), bandpass filtered (5–500 Hz) and analog-to-digitally converted at a sampling rate of 2000 Hz by PowerLab System (16/30, ADInstruments). All data were analyzed offline using Labchart seven software (ADInstruments).

### Transcranial Magnetic Stimulation

The left motor cortex was stimulated by a magnetic stimulator (Magstim 200^2^, The Magstim Company Ltd, Whitland, United Kingdom) with a 110-mm double-cone-coil (maximum output of 1.4 T). The coil was positioned to induce a postero-anterior current and manually controlled by the same investigator throughout all the testing sessions. Subjects wore a swim cap to ensure consistent coil placement relative to the optimal sites. To determine this site, six marks were drawn on the cap: the vertex, 1 and 2 cm posterior to the vertex and 1 cm to the left of these three marks along the midline. Optimal coil position was determined on the relaxed muscle as the site eliciting the largest VL, VM, and RF MEP amplitudes in response to stimulation at a given suprathreshold stimulator output (60% of maximal stimulator output). This site was marked on the swim cap and was the same for VL, VM, and RF muscles.

Resting motor threshold (rMT) was determined for the VL as the lowest TMS intensity required to elicit a MEP of minimum 50 μV peak-to-peak amplitude in at least three out of five single consecutive stimulations at that intensity from the relaxed muscle (Perez et al., 2004). The EMG signals in response to TMS were also verified to ensure that the optimal TMS stimulation intensity selected in regard to the VL rMT was able to evoke visible MEPs for both the VM and RF muscles. During the experimental session, TMS was delivered at 120% rMT, i.e., 61 ± 14% of maximal stimulator output.

### Peripheral Nerve Stimulation

Single rectangular electrical pulses with 0.2-ms duration and 400 V maximal output voltage were delivered via constant-current stimulator (DS7AH, Digitimer, Hertfordshire, United Kingdom) to the right femoral nerve via a 30-mm diameter surface cathode (Meditrace 100) taped to the skin into the femoral triangle and a 50 × 90 mm anode (Durastick Plus; DJO Global Vista, CA, United States) in the gluteal fold. To determine the optimal intensity of stimulation, single stimuli were delivered incrementally by steps of 25 mA until resting M-waves (M_max_) for VL, VM, and RF muscles plateaued. The optimal intensity was then increased by 20% to ensure supramaximality. The mean intensity for peripheral nerve stimulation (PNS) was 319 ± 129 mA.

### Local Vibration

The vibrating device (VB 115, Techno Concept, Mane, France) was locally applied and strapped directly on (i) the right RF muscle (40% of the muscle length from the upper edge of the patella to the anterior superior iliac spine) (Souron et al., 2017a,b) and (ii) the mid-portion of the infrapatellar tendon (Konishi et al., 2002; Fry and Folland, 2014) (**Figure 1B**). As Ia afferents are sensitive to a small vibration amplitude (Roll et al., 1989) and fire synchronously with vibration frequencies up to 120 Hz (Roll and Vedel, 1982; Roll et al., 1989), vibration was set in the present study with a 100-Hz frequency and 1-mm amplitude, as in our previous works on KE (Souron et al., 2017a,b). During the control condition (see the Experimental protocol section for further details), the vibrators were turned off but remained strapped to the RF and the infrapatellar tendon. Subjects were instructed to fully relax their muscles and this was verified by a direct feedback on the EMG activity during muscle and tendon LV conditions as well as during the control condition. They were also instructed to keep their eyes closed in order to prevent the occurrence of tonic vibratory reflexes induced by vision (Roll et al., 1980). Muscle inactivity was further verified by analyzing background activity as the mean value of the RMS EMG over a 100-ms period before delivery of TMS (Lapole et al., 2015b). RMS EMG was then normalized to M_max_.

### Statistics

The sample size was calculated at the outset using G^∗^Power software (version 3.1.9.2; Kiel University, Kiel, Germany) for a statistical power of 0.80 and an alpha risk of 0.05. Considering the results of several studies on upper- (Kossev et al., 1999; Siggelkow et al., 1999; Steyvers et al., 2003a) and lower-limb muscles (Lapole et al., 2015a,b), the main criterion of our study was the increase in MEPs amplitude during LV. A minimum of 20 subjects was necessary to reach the desired power for a two-way repeated-measures ANOVA. Statistical analyses were performed with Statistica software (StatSoft Inc., Tulsa, OK). All variables were normally distributed (Shapiro-Wilk normality test). For ANOVA analyses, homogeneity of variance was verified by Levene’s test. A three-way repeated measures ANOVA [condition (CON, VIB_MU_ or VIB_TD_) × angle (60, 120 or 180°) × muscle (VL, VM, or RF)] was first performed on normalized RMS values. To compare for vibration-induced changes, normalized MEPs values of each muscle recorded during VIB_MU_ and VIB_TD_ were expressed as a percentage of the CON condition. Then, a three-way repeated measures ANOVA [condition (VIB_MU_ or VIB_TD_) × angle (60, 120 or 180°) × muscle (VL, VM, or RF)] was performed to assess if one vibration condition (i.e., VIB_MU_ or VIB_TD_) or a specific muscle length (i.e., knee angle of 60, 120 or 180°) was more efficient to modulate corticospinal excitability through MEPs changes. Before that, a three-way repeated measures ANOVA [condition (CON, VIB_MU_ or VIB_TD_) × angle (60, 120 or 180°) × muscle (VL, VM, or RF)] was performed on normalized MEPs values to ensure that vibration effectively increased MEP amplitude independently of knee angle (statistics not reported). To control for possible changes in basal corticospinal excitability throughout the whole protocol, MEPs recorded before and just after the end of the protocol at a control knee angle of 120° were compared using a two-way repeated measures ANOVA (muscle × time). *Post hoc* analyses were performed using Newman-Keuls testing when the ANOVA identified significant differences. Partial eta square (*p*η^2^) was reported as an estimate of effect size, with ${\text{η}}_{\text{p}}^{2}$ ≥ 0.07 and ${\text{η}}_{\text{p}}^{2}$ ≥ 0.14 used as moderate and large effects, respectively (Cohen, 1988). Statistical significance was set at *p* < 0.05. All data are presented in the text and figures as mean ± standard deviation (*SD*).

## Results

No significant changes were found for normalized MEPs recorded before vs. after the experimental protocol for VL (2.5 ± 2.4 vs. 2.3 ± 2.2%, respectively), VM (2.9 ± 3.1 vs. 2.5 ± 2.0%), and RF (11.4 ± 8.2 vs. 12.1 ± 10.3%) muscles, as indicated by the non-significant time (*p* = 0.87; ${\text{η}}_{\text{p}}^{2}$ = 0.001) and muscle × time interaction effects interaction (*p* = 0.19; ${\text{η}}_{\text{p}}^{2}$ = 0.08). However, a significant main effect of muscle was reported (*p* < 0.001; ${\text{η}}_{\text{p}}^{2}$ = 0.52) with higher MEPs recorded on RF than VL and VM muscles.

**Table 1** presents the normalized RMS EMG for all tested muscles. There were no significant muscle × condition × angle interaction (*p* = 0.85; ${\text{η}}_{\text{p}}^{2}$ = 0.03) nor condition × angle interaction (*p* = 0.57; ${\text{η}}_{\text{p}}^{2}$ = 0.04). Similarly, no main effects of angle (*p* = 0.94; ${\text{η}}_{\text{p}}^{2}$ = 0.003) and condition (*p* = 0.52; ${\text{η}}_{\text{p}}^{2}$ = 0.03) were reported while the main effect of muscle was reported to be significant (*p* < 0.001; ${\text{η}}_{\text{p}}^{2}$ = 0.77); higher RMS EMG values were reported on RF compared to VL and VM muscles (all *p* < 0.001).

**Table 1:** Normalized EMG activity (% M_max_) before TMS of vastus lateralis (VL), vastus medialis (VM), and rectus femoris (RF) muscles with (VIB_MU_ and VIB_TD_ for muscle and tendon vibration, respectively) and without (CON) vibration.

VL			VM			RF			Knee angle
CON	VIB_MU_	VIB_TD_	CON	VIB_MU_	VIB_TD_	CON	VIB_MU_	VIB_TD_	
0.14 ± 0.08	0.14 ± 0.07	0.14 ± 0.07	0.09 ± 0.07	0.11 ± 0.08	0.11 ± 0.07	0.53 ± 0.29	0.67 ± 0.40	0.72 ± 0.62	**60°**
0.12 ± 0.05	0.12 ± 0.06	0.17 ± 0.09	0.16 ± 0.18	0.15 ± 0.11	0.18 ± 0.12	0.75 ± 0.73	0.73 ± 0.60	0.70 ± 0.61	**120°**
0.19 ± 0.24	0.21 ± 0.29	0.16 ± 0.14	0.18 ± 0.19	0.18 ± 0.18	0.14 ± 0.12	0.73 ± 0.77	0.72 ± 0.66	0.66 ± 0.59	**180°**

*Data are mean ±SD.*

**FIGURE 3:**
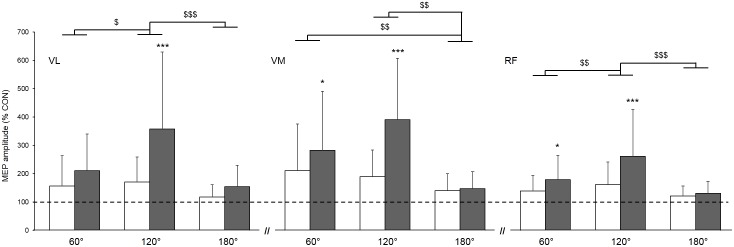
Effect of muscle (white column) and tendon (gray column) vibration on MEP amplitudes of vastus lateralis (VL), vastus medialis (VM), and rectus femoris (RF) muscles at muscle lengths corresponding to knee angles of 60, 120, and 180°. MEP amplitudes are expressed as a percentage of the mean control MEP (CON) elicited at rest without vibration. Mean control MEP (i.e., 100% on the figure) is represented by the dahsed line. Condition × angle interaction effect: ^∗^*p* < 0.05; ^∗∗∗^*p* < 0.001. Main angle effect: $*p* < 0.05; $$*p* < 0.01; $$$*p* < 0.001.

When expressing MEPs amplitudes as a percentage of the control MEP (**Figure 3**), a significant main condition effect was found, highlighting higher gains in MEPs when vibration was locally applied to the tendon compared to the muscle (*p* < 0.001; ${\text{η}}_{\text{p}}^{2}$ = 0.68); this effect was similar for all three muscles as reported by the non-significant muscle × condition interaction (*p* = 0.06; ${\text{η}}_{\text{p}}^{2}$ = 0.13). Further, a main angle effect was found (*p* < 0.001; ${\text{η}}_{\text{p}}^{2}$ = 0.44). MEP amplitudes were higher at 120° when compared to 60 and 180° and this effect was similar for all of the three muscles as reported by the non-significant muscle × angle interaction (*p* = 0.13; ${\text{η}}_{\text{p}}^{2}$ = 0.08).

## Discussion

The main aim of this study was to investigate the effects of (i) muscle length and (ii) vibration site on corticospinal excitability during LV of the KE. In agreement with our hypothesis, the main finding is that MEPs is greater when LV is applied to the tendon compared to the muscle. On the opposite, the hypothesis that the increase in MEPs amplitude during LV would be higher on stretched muscle is not confirmed since a greater increase in MEPs amplitude was reported at an intermediate muscle length (corresponding to a knee angle of 120°) compared to short (knee angle of 180°) or long (knee angle of 60°) muscle lengths. Still, it shows that muscle length plays a role on corticospinal excitability.

It is now well accepted that activation of Ia afferents from muscle spindles during LV increases corticospinal excitability as indicated by increased MEPs size during LV in various upper- (Kossev et al., 1999, 2001; Siggelkow et al., 1999;Rosenkranz et al., 2003; Steyvers et al., 2003a) and lower-limb muscles (Lapole et al., 2015a,b). The present study further reports such findings for the KE and confirms the potential for LV to modulate corticospinal excitability of lower-limb muscles, despite weaker corticomotoneuronal projections compared with upper-limbs (Brouwer and Ashby, 1990). One may only speculate about the mechanisms involved in the LV-induced increased MEPs amplitude since MEPs depends on the synaptic relays of the corticospinal projections at both the cortical and spinal levels (Devanne et al., 1997). Although cortical mechanisms have been suggested to increase corticospinal excitability during LV (Kossev et al., 1999), potentially through long-loop reflexes involving transcortical circuits (Matthews, 1984), we previously reported increased soleus MEPs during LV without concomitant changes in intracortical inhibition and facilitation suggesting that increased corticospinal excitability would be rather spinal in origin (Lapole et al., 2015a). Hence, vibration-induced Ia afferent activity may have partially depolarized motoneuronal cells without causing them to discharge, as confirmed by the unaltered EMG background activity reported during vibration. Thus, motoneurons would have been more responsive to subsequent TMS. Such LV-induced modulations in motoneuronal excitability has been recently confirmed by our team after a 30-min prolonged LV exposure on KE (Souron et al., 2017b) but remains to be determined during LV. However, this remains purely speculative and the absence of spinal excitability measurements in the present study is a limitation of the present work. Future studies combining TMS and PNS measurement (e.g., H reflex, cervicomedullary and/or thoracic motor evoked potentials) should be conducted to give more precise insights into potential mechanisms for enhanced MEPs during LV by further distinguishing cortical and spinal excitability.

Changes in MEPs during LV may thus document the efficiency for LV to strongly activate Ia afferents. The LV-induced acute and chronic effects are directly related to the activation of muscle spindle primary afferents [see Souron et al. (2017c) for a review]. Thus, whether or not changes in vibration characteristics induced different responses in MEPs during LV may help to characterize optimal parameters for both acute and chronic LV interventions. In addition of vibration frequency (Siggelkow et al., 1999; Steyvers et al., 2003a; Lapole et al., 2015b), other variables remain to be investigated to determine optimal vibration characteristics. This paper focused on vibration site and muscle length.

### Influence of Vibration Site

When considering the acute effects of LV on the KE, vibration has been locally applied either on the muscle (Kouzaki et al., 2000; Jackson and Turner, 2003; Iodice et al., 2011; Saito et al., 2016b; Souron et al., 2017b) or the tendon (Konishi et al., 2002, 2009; Richardson et al., 2006; Fry and Folland, 2014; Saito et al., 2016a). While both VIB_MU_ and VIB_TD_ were effective to increase KE corticospinal excitability in this study, higher increases in MEPs amplitude were found for VIB_TD_ compared to VIB_MU_ for VL (mean pooled increase for the three angles: + 241 vs.+ 148% for VIB_TD_ and VIB_MU_, respectively), VM (+273 vs. 180%) and RF muscles (+191 vs. +141%). This suggests a greater facilitatory effects at the spinal and/or cortical level, which could be related to a greater discharge of Ia afferents when vibration is locally applied to the tendon rather than the muscle. While the tonic vibration reflex is not always observed when the vibration is applied to the muscle, it is usually detected when the vibration is applied to the tendon (De Gail et al., 1966; Eklund and Hagbarth, 1966). Moreover, it seems that less responsive endings (e.g., type II afferents) discharge only when vibration is locally applied to the tendon (Burke et al., 1976b), suggesting that the activation of endings other than the Ia afferents by tendon vibration may have led to a greater modulation in corticospinal excitability. Further investigations using microneurographic recordings (Roll et al., 1989) would help in elucidating the neurophysiological mechanisms explaining the greater increase in MEPs during VIB_TD_.

### Influence of Muscle Length

Various muscle lengths have been used in studies that assessed the acute effects of LV to modulate neuromuscular function, i.e., knee angle of 75° (Richardson et al., 2006), 90° (Kouzaki et al., 2000; Konishi et al., 2002, 2009; Jackson and Turner, 2003; Saito et al., 2016b,a; Souron et al., 2017b), 120° (Fry and Folland, 2014), and 180° (Iodice et al., 2011). Similarly, studies that investigated the effect of LV as a training method on the KE used various muscle lengths; some have applied LV at shortened muscle length, i.e., knee angle at 180° (Iodice et al., 2011; Tankisheva et al., 2015) while others used intermediate muscle length, i.e., knee angle at 90° (Souron et al., 2017a). The initial muscle state at which LV is applied is another parameter to consider since it significantly influences muscle spindles sensitivity (Vallbo, 1974; Burke et al., 1976b, 1978). In the present study, three muscle lengths were tested, corresponding to short/intermediate/long quadriceps muscle length. We found that the increase in MEPs amplitude was higher at an intermediate (mean pooled increase for VIB_TD_ and VIB_MU_: +265, +290, and +212% for VL, VM, and RF, respectively) compared to short (+136, +144, and +127%) or long (+184, +246, and +160%) muscle lengths. To the best of our knowledge, only one study (Burke et al., 1976b) has investigated the effects of muscle length on LV responses. It was found that tibialis anterior muscle spindles sensitivity was increased (i.e., higher Ia afferents discharge in response to LV) when the muscle was slightly lengthened (i.e., 7° of plantarflexion) when compared to a neutral position corresponding to an ankle angle of 90°. This is in agreement with the greater KE MEPs amplitude reported in the present study at an intermediate quadriceps length compared to a shortened quadriceps length, the intermediate length being slightly above the slack length for quadriceps muscles, i.e., around 140° which may be considered as the neutral knee angle (Xu et al., 2016). We speculate that the lower MEPs amplitude reported at long vs. intermediate muscle length during LV could be due to a “busy-line” phenomenon (Hagbarth, 1973). This author suggested that the suppression of tendon jerks, phasic stretch and H-reflexes commonly reported during LV could be explained by the fact that Ia afferents were so activated by the vibratory stimulus that they were not able to respond efficiently to a transient superimposed muscle stretch. The LV-induced afferent inflow leading to reflexes suppression may also be influenced by spinal presynaptic inhibition of the monosynaptic pathway involving GABA_A_ neurotransmission (Hagbarth, 1973). In the present study, it is likely that Ia afferents already discharged when the quadriceps was placed at a long length without the presence of any vibration stimulus (Burke et al., 1976b) so that a “busy-line” effect could have prevented the discharge of the Ia afferents to substantially increase in response to LV (Hagbarth, 1973; Bove et al., 2003). Alternatively, it may also be speculated that the activity from Golgi tendon organs Ib afferents may have contributed to the lower increase in MEPs amplitude reported in this study at long vs. intermediate muscle length during LV by reducing motoneuron output (Jami, 1992) through inhibitory postsynaptic mechanisms (Duchateau and Enoka, 2016). For instance, Ib afferents are sensitive to muscle tension, not only during muscle contraction but also in response to muscle stretch (Stuart et al., 1970), and have been reported to be more sensitive to vibration at long muscle length (Burke et al., 1976b). Then, it is possible that Ib afferents discharge increased when the muscle was placed at a long muscle length corresponding to a knee angle of 60° in the present study, which could have dampened the expected vibration-induced Ia excitation effect on MEPs amplitude.

## Conclusion

This study focused on the influence of vibration site and muscle length on corticospinal excitability modulations. It was found that the greatest MEPs were obtained when vibration was locally applied to the tendon at an intermediate muscle length. Based on the present results and the findings of other authors about the influence of vibration frequencies (Siggelkow et al., 1999; Steyvers et al., 2003b; Lapole et al., 2015b), we suggest that the following LV characteristics must be used to optimize the effects of LV on the neuromuscular function, at least for knee extensor muscles: (i) a vibration frequency within the optimal range of Ia afferents discharge, i.e., 50–120 Hz; (ii) applied to the tendon rather than the muscle; (iii) at an intermediate muscle length – corresponding to 120° for the knee joint (180° being full knee extension). Future studies should investigate the role of other parameters such as the optimal duration of the LV application to induce immediate and persistent modulations of neuromuscular function.

## Author Contributions

RS was involved in the conception of the research, the acquisition, analysis and interpretation of the data. RS was also involved in the revision of the manuscript. MO was involved in the conception of the research, the acquisition, analysis, and interpretation of the data, as well as in the revision of the manuscript. GM and TL were involved in the conception of the research design, the analysis and interpretation of the results and the writing of the manuscript (revising the work). As the contributing author, TL gave his final approval for submission.

## Conflict of Interest Statement

The authors declare that the research was conducted in the absence of any commercial or financial relationships that could be construed as a potential conflict of interest. The reviewer KP and handling Editor declared their shared affiliation at the time of the review.
